# Integrative Analysis of miRNA and mRNA Expression Profiles in Calcium Oxalate Nephrolithiasis Rat Model

**DOI:** 10.1155/2017/8306736

**Published:** 2017-12-17

**Authors:** Chuangxin Lan, Dong Chen, Xiongfa Liang, Jian Huang, Tao Zeng, Xiaolu Duan, Kang Chen, Yongchang Lai, Dong Yang, Shujue Li, Chonghe Jiang, Wenqi Wu

**Affiliations:** ^1^Department of Urology, Minimally Invasive Surgery Center, The First Affiliated Hospital of Guangzhou Medical University, Guangzhou, Guangdong, China; ^2^Guangzhou Institute of Urology, Guangzhou, Guangdong, China; ^3^Guangdong Key Laboratory of Urology, Guangzhou, Guangdong, China; ^4^Department of Urology, Huizhou Municipal Central People's Hospital, Huizhou, Guangdong, China; ^5^Department of Urology, Qingyuan People's Hospital, The Sixth Affiliated Hospital of Guangzhou Medical University, Qingyuan, Guangdong, China

## Abstract

The microRNA (miRNA) expression profiles and their biological functions in calcium oxalate nephrolithiasis remain unclear. In this study, we investigate the miRNA and mRNA expression profiles of kidney tissues in calcium oxalate stone rats. 16 Sprague Dawley rats were divided into control group and stone-forming group. 24-hour urine samples and kidney tissues were collected for biochemical and histological determination after 4 weeks. MiRNA and mRNA microarray were applied to evaluate the miRNA and mRNA expression profiles. To validate the microarray results, the quantitative real-time PCR (qRT-PCR) was performed. A total of 38 miRNAs and 2728 mRNAs were significantly and differentially expressed in kidney tissues of stone-forming group versus control group. Gene Ontology (GO) analysis revealed that most of the target genes were enriched in terms of oxidation reduction, ion transport, inflammatory response, and response to wounding. Kyoto Encyclopedia of Genes and Genomes (KEGG) pathway analysis of these targets highlights their critical role in cytokine-cytokine receptor interaction, gap junction, and chemokine signaling pathway. Furthermore, the reliability of the microarray-based results was confirmed by using qRT-PCR determination. The miRNA and mRNA expressions in calcium oxalate stone rat kidneys might provide a basis for further research on urolithiasis mechanism.

## 1. Introduction

Urolithiasis is a worldwide disease with high morbidity and economic costs. The kidney stone prevalence in US showed a linear increase over the past four decades, that is, from 3.8% during 1976–1980 to 8.8% during 2007–2010 [1, 2]. It has been reported that the prevalence in China was 6.50% [3]. More than half of the stone patients experience a recurrence within 5–10 years of a first episode [4]. Calcium oxalate, the primary constituent of urolithiasis, accounts for approximately 80% of the total urinary tract stone [5], whose pathogenesis remains a mystery though existing theories showed that it was a multifactorial disorder including genetic, metabolic, and dietary factors [6].

MicroRNAs (miRNAs), a class of endogenous noncoding small RNA molecules of 21–23 nucleotides, play a crucial regulatory role in degrading target mRNAs or repressing their translation by binding to complementary sites on target mRNAs in the 3′UTR [7]. In eukaryotic cells, miRNAs can regulate 31% of the target genes and participate in multiple processes of biological development and diseases [8]. Recent studies implicated that up- or downregulation of certain miRNAs might play a major role in the cause of hypercalciuria, which is an important risk factor for calcium urolithiasis [9]. However, there is a paucity of research about miRNA expression patterns and their biological functions in urolithiasis, which seems important to further understand the relationship between genes and pathophysiological process of urolithiasis.

In this study, we applied miRNA, mRNA microarray technique, and bioinformatics databases to integrally analyze the miRNA and mRNA expression profiles of kidney tissues in calcium oxalate stone rats, aiming at exploring more specific and effective molecular targets for urolithiasis.

## 2. Materials and Methods

### 2.1. Animal Model of Urolithiasis

Hyperoxaluria is one of the most common risk factors of calcium oxalate nephrolithiasis, which can be induced by sustained administration of oxalate or its precursors such as ethylene glycol (EG). During administration of EG with ammonium chloride (AC), the urinary excretion of oxalate increased rapidly and calcium oxalate supersaturation increased accordingly. Then crystalluria was produced and was followed by calcium oxalate nephrolithiasis [10]. In the present study, a total of 16 male Sprague Dawley rats (171 ± 5.2 g) were utilized and purchased from Guangdong Medical Laboratory Animal Center, China. They were kept in standard laboratory conditions (under natural light and dark cycles) and received food and water ad libitum before the experiment. 16 rats were randomly divided into two groups of 8 rats each. In the control group, rats were fed with free access to food and normal drinking water, while, in the stone-forming group, rats were fed with free access to food and drinking water containing 1% EG for 4 weeks. Besides, 1% AC was given by gavage for 5 days at the early stage of experiment. The experimental protocol was approved by the Ethics Review Board of Guangzhou Medical University, Guangzhou, China.

### 2.2. Collection of Urine and Kidney Specimen

The duration of the experiment was 4 weeks. 24-hour urine samples were collected on the day before the end of the experiment. At the end of the experimental period, all the rats were sacrificed by overdose of pentobarbital sodium (50 mg/kg, i.p.) and then both sides of the kidneys were removed after sufficient transcardial perfusion. One kidney was stored in liquid nitrogen for molecular biological studies and the other was fixed in 4% paraformaldehyde for histological studies.

### 2.3. Biochemical Determinations of Urine and Histological Studies

The urine oxalate and citrate were measured using an ion chromatograph (883 Basic IC plus, Metrohm AG, Switzerland) while the urine calcium was determined by the automatic biochemical analyzer. Rat kidney tissues were embedded in paraffin. Cross-sections of 5 *μ*m were prepared and stained with hematoxylin/eosin (HE). Then the tissues and crystals were examined using polarizing microscope.

### 2.4. RNA Extraction

Total RNA was extracted from kidney cortex tissue adjacent to stones using Trizol Reagent (Ambion, USA) according to the manufacturer's protocol. The purity and concentration were assessed by NanoPhotometer (Implen, Germany).

### 2.5. Microarray Analysis

Three stone-forming rats and three control rats were randomly selected for microarray analysis. The Agilent Rat miRNA Microarray Release 19.0 and Agilent Rat Microarray 4*∗*44 K were used to compare miRNA and mRNA expression profiles, respectively, in both control and stone-forming rats. Microarray analyses were performed by CapitalBio Corporation (Beijing, China) and Biotechnology Corporation (Shanghai, China), respectively.

### 2.6. Real-Time RT-PCR

For miRNA analysis, total RNA was used for cDNA synthesis and quantitative detection according to the protocol of the All-in-One™ miRNA qRT-PCR Detection Kit (GeneCopoeia, Guangzhou, China). For mRNA analysis, total RNA was reverse-transcribed to cDNA using Takara reverse transcription kit (Takara, Dalian, China). PCR was conducted according to standard protocol of SYBR Premix Ex Taq (Takara, Dalian, China). The primers were chemically synthesized by Shanghai Generay Biotech Co., Ltd. U6 and GAPDH were used as the internal controls of miRNA and mRNA, respectively. All reactions were performed in triplicate.

### 2.7. Target Prediction and Function Analysis

Five bioinformatics databases, including TargetScan, miRanda, miRDB, miRWalk, and PITA, were utilized to predict the potential target genes. Genes present in three of the five databases were considered as potential target mRNAs. Then these genes were compared with those from mRNA microarray, and the overlaps of them were determined. As miRNA exerts its biological functions through the suppression of target genes, that is, miRNA negatively modulates gene expression, we intersected the predicted target mRNAs of upregulated miRNAs with the downregulated mRNAs from microarray, and vice versa. In order to determine the biological functions and pathways that were potentially functional in the formation of kidney stone, we performed Gene Ontology (GO) and Kyoto Encyclopedia of Genes and Genomes (KEGG) annotations of the predicted target mRNAs by DAVID Bioinformatics Resources.

### 2.8. Statistical Analysis

Continuous variables were expressed as means ± standard deviation; then Student's *t*-test was applied for statistical analysis by using SPSS 13.0. The raw data of microarray were analyzed by using GeneSpring and the RVM (random variance model) *t*-test was performed to filter the differentially expressed miRNAs/mRNAs between the two groups [11]. A *P* value of <0.05 was considered to be statistically significant.

## 3. Results

### 3.1. Urine Biochemical Analysis and Histological Observation

**Figure 1:**
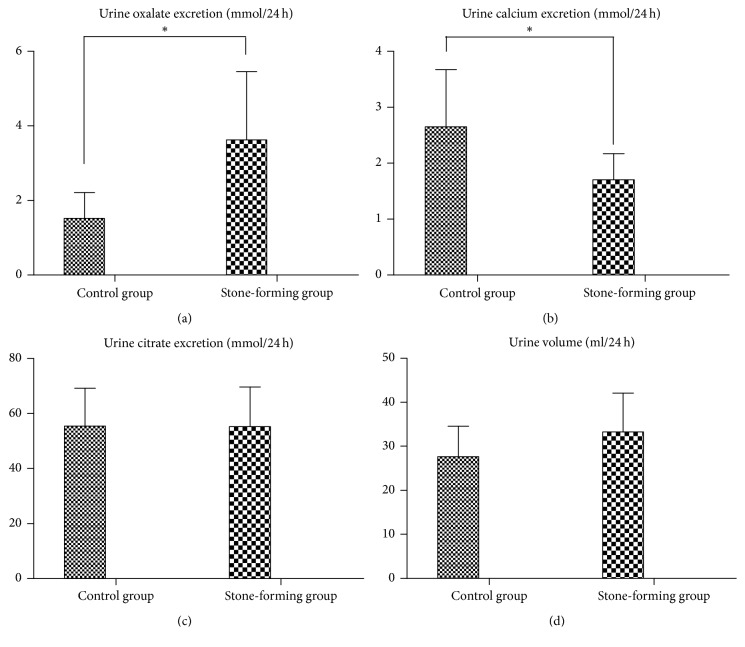
Comparisons of biochemical analysis of urine between stone-forming and normal rats. The 24-hour urine oxalate (a) was significantly increased in stone-forming rats compared with control rats, while the 24-hour urine calcium (b) was decreased. The 24-hour urine citrate (c) and volume (d) were not significantly altered. ^*∗*^*P* < 0.05 versus control.

Comparisons of biochemical analysis of urine between two groups of rats are illustrated in Figure 1. After a four-week ethylene glycol administration, the average urine oxalate excretion in stone-forming rats was significantly enhanced compared to the control rats (3.63 ± 1.83 versus 1.52 ± 0.69 mmol/24 h), whereas urinary calcium was significantly reduced (1.70 ± 0.47 versus 2.65 ± 1.02 mmol/24 h). Urine citrate and volume, however, were not significantly altered in the two groups.

**Figure 2:**
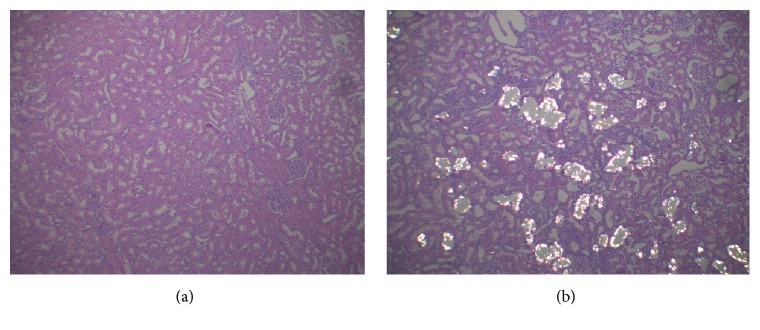
Histological observation of crystals in rat kidney. No crystals were observed in control group (a), but, in stone-forming group (b), crystals were observed in the renal tubular lumen.

The histological changes of kidney sections in the two groups of rats are shown in Figure 2. In stone-forming group, the crystal deposits were displayed in the renal tubular lumen of cortex and medulla region and were irregular in shape and size. Moreover, most of the renal tubules were distended. By contrast, the kidney of control group showed normal morphology and no crystals were observed in them at all.

### 3.2. Alteration of miRNA and mRNA Expression Profiles

The miRNA expression profiles were significantly different in rat kidney tissues between the two groups. Of all the miRNAs measured, 38 mature miRNAs were identified. In the rats of stone-forming group, the deferential expressions revealed 2-fold changes compared to that in the rats of control group (*P* < 0.05), including 18 upregulated miRNAs and 20 downregulated ones. Among these differentially expressed miRNAs, rno-miR-130b-3p, rno-miR-132-3p, rno-miR-181a-1-3p, rno-miR-222-3p, rno-miR-351-5p, and rno-miR-21-3p had the largest positive fold changes, while rno-miR-335, rno-miR-192-3p, rno-miR-194-5p, rno-miR-192-5p, rno-miR-499-5p, and rno-miR-210-3p had the largest negative fold changes (Table 1).

In order to investigate potential targets of the altered miRNAs, we also determined the mRNA expression profiles of the same kidney tissues by using Agilent microarray. The results showed that, among the 2728 differentially expressed mRNAs, 1350 mRNAs were significantly increased whereas 1378 mRNAs were significantly decreased in stone-forming group. The top 10 upregulated genes were Il19, Reg3b, Havcr1, C4bpa, Mmp7, Il24, Fgg, Fgb, Gpnmb, and Cxcl2. Among the downregulated genes, the top 10 were Cyp2c11, LOC102550988, RGD1306462, Serpinb12, LOC361914, Cyp1a1, Cacng5, Iqch, Pkd2l2, and LOC691551 (Table 2).

### 3.3. miRNA Target Gene Prediction

As miRNAs negatively regulate mRNAs expression via degradation or translation inhibition, we intersect the predicted target mRNAs of upregulated miRNAs with the downregulated mRNAs from microarray, and vice versa. Among the 1378 significantly decreased mRNAs, 141 genes were the predicted targets of the 18 upregulated miRNAs. Similarly, 187 in 1350 overexpressed mRNAs and 20 downregulated miRNAs were predicted as targets. According to the above method, a total of 797 significant miRNA-mRNA pairs were predicted, consisting of the 38 differentially expressed miRNAs and 328 mRNAs. The predicted targets of these miRNAs were then used for downstream GO analysis and KEGG pathway analysis.

### 3.4. GO and KEGG Pathway Analysis

Functional enrichment analysis of the target genes of the differentially expressed miRNAs was performed by means of Gene Ontology system, and a few representative biological processes were revealed in Table 3. According to the GO analysis, there were 362 GO terms significantly enriched (*P* < 0.05). The most highly enriched biological function of the upregulated miRNAs included oxidation reduction (GO:0055114), response to hormone stimulus (GO:0009725), and ion transport (GO:0006811), while response to wounding (GO:0009611), inflammatory response (GO:0006954), and defense response (GO:0006952) were associated with the downregulated miRNAs.

Potential functional pathways for the targets of differentially expressed miRNAs were then predicted by KEGG pathway system. Based on the KEGG pathway analysis, 19 pathways were predicted to be significantly related to stone formation (*P* < 0.05). Cytokine-cytokine receptor interaction, gap junction, and chemokine signaling pathway were the main pathways involved in these predicted pathways (Table 4).

### 3.5. Validation of the Microarray Expression Data by Real-Time PCR

**Figure 3:**
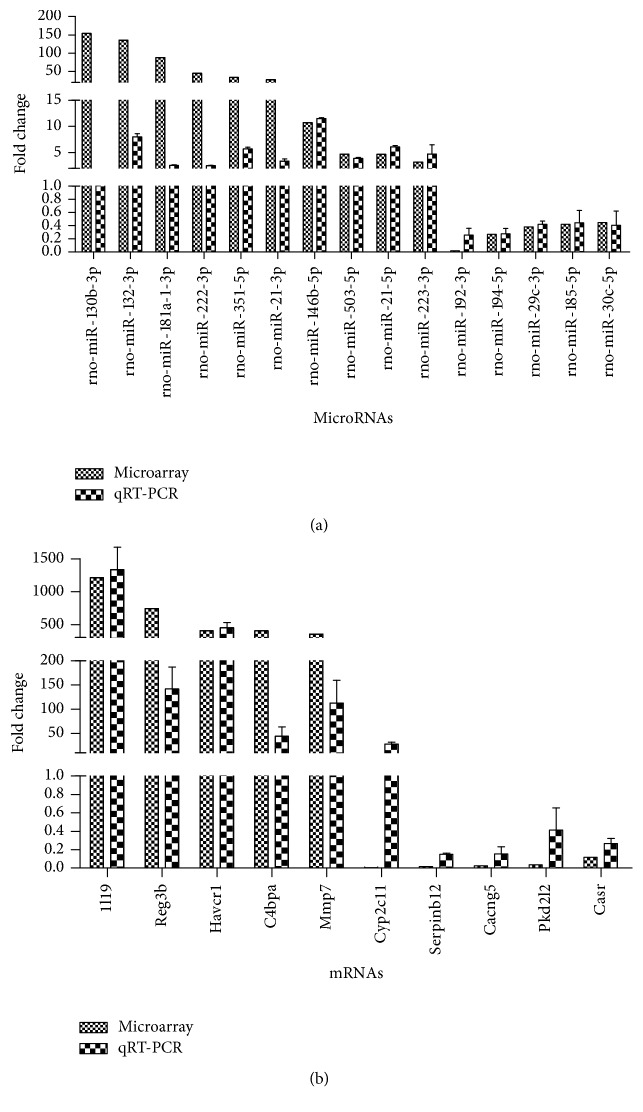
qRT-PCR validation of differentially expressed miRNAs and mRNAs. Quantitative reverse transcription PCR was performed to confirm the expression of 15 select miRNAs (a) and 10 mRNAs (b).

To validate the reliability of our microarray-based results, qRT-PCR was performed in 5 pairs of matched stone-forming/control rat kidneys. From the differentially expressed miRNAs and mRNAs observed by microarray, 15 miRNAs and 10 mRNAs were selected. Relative expression levels of the selected miRNAs and mRNAs were depicted in Figure 3. Consistent with the microarray data, real-time PCR confirmed that, compared with controls, rno-miR-132-3p, rno-miR-181a-1-3p, rno-miR-222-3p, and rno-miR-351-5p were significantly increased, while rno-miR-192-3p, rno-miR-194-5p, rno-miR-29c-3p, rno-miR-185-5p, and rno-miR-30c-5p were significantly decreased in stone-forming rat kidneys. Besides, the expressions of Il19, Reg3b, Havcr1, C4bpa, and Mmp7 were significantly increased, and those of Serpinb12, Cacng5, Pkd2l2, and Casr were significantly decreased in stone-forming group, which is in line with the microarray data, too. Although the magnitude of changes differed between the two methods, these results demonstrated a high consistency between the microarray and RT-qPCR based results except for rno-miR-130b-3p and Cyp2c11.

## 4. Discussion

In the present study, we found 38 miRNAs and 2728 mRNAs differentially expressed in kidney tissues between calcium oxalate stone-forming rats and the controls. 328 potential target genes for the 38 miRNAs were predicted by intersecting five bioinformatics databases and differentially expressed mRNAs, resulting in 797 miRNA-mRNA pairs. GO term and KEGG pathway analysis further revealed that the miRNA-mRNA contributed a lot to the calcium oxalate stone formation. In addition, the expression of several miRNAs and mRNAs was confirmed via qRT-PCR and the outcomes were highly consistent with the microarray data, indicating that our microarray-based determinations were reliable.

As posttranscriptional gene regulators, miRNAs are involved in diverse pathophysiological processes of various diseases [8]. There are growing evidences that miRNAs play a vital role in the formation of kidney stone. Hu et al. [12] reported that the miR-155 level in serum and urine was significantly increased in patients with nephrolithiasis and proposed that miR-155 might influence the nephrolithiasis pathophysiological process by regulating inflammatory cytokines expression. Though there was no difference of miR-155 in the expression between the two groups of our study, we found that the targets of miRNAs were enriched in the biological function of inflammatory response. Another study also reported that miRNAs were significantly differently expressed in proximal renal tubular cells due to exposure to calcium oxalate monohydrate crystal and some of them might be involved in the insulin signaling pathway and type 2 diabetes mellitus [13].

Recently, Liu and his colleagues [14] established a hyperoxaluric rat model induced by feeding of ethylene glycol and ammonium chloride and demonstrated that the target genes and signaling pathways were involved in the pathogenesis of hyperoxaluria. They showed that 28 miRNAs were differentially expressed between the experimental and control groups. In addition, the GO and KEGG analyses revealed that these miRNAs were potentially associated with the insulin resistance and phosphatidylinositol-bisphosphonate 3-kinase/AKT serine threonine kinase signaling pathways. Among the miRNAs differentially expressed in Liu's study, rno-miR-132-3p, rno-miR-146b-5p, rno-miR-223-3p, rno-miR-21-5p, and rno-miR-214-3p were upregulated, which was just the same as our findings. However, their rat models were created by free access to water containing 1% EG and 0.5% AC for two weeks. Since kidney stone is a chronic disease, 2 weeks of modeling time might be not conformable with regard to the natural course of stone formation. In addition, AC was reported to have nephrotoxicity, which might cause kidney injury and in consequence affect the outcomes of the study when used for a longer time. Therefore, our rat models were induced by drinking water with 1% EG for 4 weeks, and 1% AC was given as gavage during the early 5 days. Theoretically, this model would be more convincing and the obtained data more reliable.

Lu et al. [9] reported that there were 19 miRNAs significantly expressed in genetic hypercalciuric stone-forming rat kidneys compared to normal kidneys, including 10 upregulated miRNAs and 9 downregulated ones. Among them, rno-miR-674-5p, rno-miR-672-5p, rno-miR-138-1-3p, and rno-miR-21-3p were significantly expressed and might play important roles in the regulatory network. KEGG pathway analysis showed that NF-*κ*B signaling pathway was significantly enriched in target genes of differentially expressed miRNAs, suggesting that it might be associated with hypercalciuria urolithiasis. Out of the 19 reported miRNAs that were dysregulated, miR-21-3p was the only one miRNA identified as the upregulated gene in the above-mentioned two studies.

With respect to oncogenic miRNA, miR-21 was traditionally regarded as highly associated with variety of tumors, such as breast cancer, ovarian cancer, or urothelial carcinoma [15–17]. Recent study suggested that miR-21 might participate in the development of fibrosis via promoting the proliferation of interstitial fibroblasts and increasing the abnormal deposition of the extracellular matrix [18]. Other investigators showed that miR-21 could inhibit CD40 expression through SIRT1-NF-*κ*B signaling, which might negatively regulate inflammatory processes in renal tubule epithelial cell [19–21].

Moreover, miR-21 was regarded as modulatory reaction of oxidative stress since the SOD expression was decreased and ROS formation increased [22]. It prompts us to believe that, no matter whether mediated by hypercalciuria or hyperoxaluria, miR-21 was the common miRNA that was overexpressed in the process of stone formation, which might subsequently cause oxidative stress or stimulate renal tubular epithelial cells apoptosis and tubulointerstitial fibroblast proliferation, thus resulting in the fibrotic dysfunction of kidney by certain downstream signaling pathways such as PTEN/Akt pathway [23]. Therefore, miR-21 might be crucial in the processes of stone formation, and the mechanism needs to be further investigated.

Based on the GO analysis, the most highly enriched biological functions of dysregulated miRNAs targets are oxidation reduction, ion transport, response to wounding and inflammation, and so on. Similar study showed that oxidative stress was intimately involved in signaling molecule changes, renal tubular epithelial cell injury, and inflammation during the renal stone formation [6, 24, 25]. The oxidants such as ROS could severely affect the cells structure and function in consequence of reacting with all the cell basic constituents [26]. Both in vivo and in vitro animal experiments demonstrated that ROS could generate the formation of kidney stone, and treatment with antioxidants significantly reduces the crystal deposition [27–29]. Obviously, the crystal-associated pathological changes in kidney resulted from the damage effects of oxidants and from the oxidants-mediated changes in gene expression and signal transduction.

Inflammation is a complex biological response to various irritants and inflammatory cells. The inflammatory cells, such as monocyte and macrophages, are usually found to migrate to the sites of crystal deposition, which is probably due to the effects of inflammatory cytokines, chemokines, and adhesion molecules released by renal tubular epithelial cells [21, 30]. Normally, once crystals have been formed, they would be soon cleared away by the protective response, that is, producing macromolecules and phagocytose to eliminate them, rendering them harmless [31]. In agreement, we showed that the expression of rno-miR-192, rno-miR-194, and rno-miR-499 was higher in the stone-forming group while their potential target gene chemokine receptor 2 (CCR2) was lower. Another study demonstrated that CCR2, the receptor of chemoattractant protein-1 (MCP-1), could stimulate macrophages infiltration, tubulointerstitial injury, and fibrosis, which was closely associated with stone formation [32]. Therefore, we proposed that, in the process of kidney stone formation, the overexpression of CCR2 mediated by relevant miRNAs, such as rno-miR-192, rno-miR-194, and rno-miR-499, would induce the inflammation and damage to the renal tubular epithelial cells and promote nephrolithiasis. Ion transport is another major pathogenesis for the formation of nephrolithiasis. In this study, we showed that rno-miR-223-3p was upregulated whereas CaSR was downregulated in the kidney of stone-forming group, indicating that CaSR was a potential target gene of rno-miR-223-3p. Recent research showed that the expression of CLDN14 was increased in mice with high dietary calcium intake. It was assumed that this regulation might be via the activation of CaSR, blockage of the paracellular reabsorption of Ca^2+^, and facilitation of the renal Ca^2+^ losses [33–35]. Therefore, the disturbance of this signaling pathway might contribute to calcium changes by renal calcium excretion, which is closely associated with stone formation.

In addition, as predicted by the bioinformatics databases, SLC4A1 was the potential target gene of rno-miR-34b, rno-miR-146b, rno-miR-214, rno-miR-223, and rno-miR-351. SLC4A1, which encoded anion exchanger-1 (AE1), was manifested as bicarbonate reabsorption and urinary acidification [36]. SLC4A1 was differentially downregulated in stone-forming group in our study. Previous reports suggested that the abnormal function of SLC4A1 was a risk factor for idiopathic calcium nephrolithiasis, and the mutations of it might cause distal renal tubular acidosis, which also promoted recurrent renal stone formation [37, 38]. Therefore, in the process of stone formation, the upregulation of these miRNAs might downregulate the expression of SLC4A1, which impacted the acid-base homeostasis and led to stone formation.

In previous studies, pathways such as p38 MAPK pathway and TGF*β* pathway had been reported to be related to the process of stone formation [28, 39]. However, our results did not draw the same conclusion. The KEGG pathway analysis indicated that cytokine-cytokine receptor interaction and chemokine signaling pathway were significantly enriched in stone-forming rats. It seems that KEGG pathways are consistent with the GO terms because both of them were involved in the development of inflammation.

The findings of the present study may provide novel biomarkers for diagnosis, prognosis, and clinical trials of calcium oxalate nephrolithiasis. However, as the urine calcium excretion was lower in the stone-forming group, we are not so sure whether the miRNAs and mRNAs observed in this animal stone model are consistent with the natural pathogenesis of human stone disease. Other limitations of this study are whether the altered mRNAs could be translated into the similar proteins and what the exact target gene and biological function of these dysregulated miRNAs are. All these issues need further study for clarification.

## 5. Conclusion

In summary, the identification of differentially expressed miRNAs and mRNAs in the kidneys between calcium oxalate stone-formation rats and normal ones was determined. Some pathway analysis in this study, such as cytokine-cytokine receptor interaction, gap junction, and chemokine signaling pathway, provides a basis and new direction for further research on urolithiasis mechanism.

## Figures and Tables

**Table 1:** Total dysregulated miRNAs between stone-forming and normal rats.

Upregulated miRNAs	Fold change	*P* value	Downregulated miRNAs	Fold change	*P* value
rno-miR-130b-3p	153.93	0.0033	rno-miR-335	92.07	0.038
rno-miR-132-3p	135.68	0.016	rno-miR-192-3p	56.46	0.036
rno-miR-181a-1-3p	88.12	0.0033	rno-miR-194-5p	3.75	0.011
rno-miR-222-3p	44.97	0.012	rno-miR-192-5p	3.68	0.012
rno-miR-351-5p	33.89	0.0033	rno-miR-499-5p	2.98	0.023
rno-miR-21-3p	28.02	0.043	rno-miR-210-3p	2.89	0.041
rno-miR-146b-5p	10.73	0.0068	rno-miR-200b-5p	2.88	0.030
rno-miR-503-5p	4.71	0.0011	rno-miR-347	2.88	0.048
rno-miR-21-5p	4.68	0.011	rno-miR-29c-3p	2.64	0.00048
rno-miR-34b-5p	4.03	0.016	rno-miR-29c-5p	2.56	0.0032
rno-miR-542-5p	3.62	0.039	rno-miR-185-5p	2.39	0.013
rno-miR-223-3p	3.21	0.012	rno-miR-218a-5p	2.38	0.012
rno-miR-18a-5p	2.98	0.038	rno-miR-378a-5p	2.34	0.0033
rno-miR-214-3p	2.83	0.024	rno-miR-378b	2.27	0.011
rno-miR-542-3p	2.65	0.015	rno-miR-30e-3p	2.26	0.0033
rno-miR-199a-5p	2.58	0.016	rno-miR-30c-5p	2.24	0.0042
rno-miR-322-5p	2.42	0.014	rno-miR-30e-5p	2.23	0.014
rno-miR-142-3p	2.29	0.011	rno-miR-203a-3p	2.17	0.036
			rno-miR-200a-3p	2.10	0.0033
			rno-miR-429	2.01	0.0042

**Table 2:** Top 10 up- and downregulated mRNAs between stone-forming and normal Rats.

Upregulated mRNAs	Fold change	*P* value	Downregulated mRNAs	Fold change	*P* value
Il19	1212.01	0.00028	Cyp2c11	307.34	0.0018
Reg3b	739.23	1.5*E* − 06	LOC102550988	118.35	0.0065
Havcr1	404.69	1.7*E* − 05	RGD1306462	69.72	0.0083
C4bpa	402.77	0.0065	Serpinb12	67.62	0.0063
Mmp7	354.62	0.0047	LOC361914	54.3	0.0065
Il24	242.97	0.0036	Cyp1a1	45.19	0.00095
Fgg	187.73	0.00050	Cacng5	44.06	0.00011
Fgb	179.11	3.3*E* − 05	Iqch	37.51	0.00064
Gpnmb	139.07	0.00035	Pkd2l2	29.50	0.0063
Cxcl2	105.48	0.0067	LOC691551	29.27	0.0074

**Table 3:** Representative GO terms of the predicted miRNAs targets.

GO ID	GO term	Target genes	*P* value
*Upregulated miRNAs*			
GO:0055114	Oxidation reduction	ME1, BCKDHA, LDHC, SUOX, GPD1, ADHFE1, CYP1A1, HSD3B6, GLUD1, CYCS, HGD, GCLM, HIBADH, ACOX3, FDFT1, IVD, DHCR7, HSD11B1, AKR7A3, DMGDH, MECR, ALDH9A1, NQO2	5.7*E* − 08
GO:0009725	Response to hormone stimulus	ME1, BCKDHA, AR, CRYAB, STAT5A, GGH, AQP1, PPARGC1B, GHRHR, PRSS8, EIF4EBP2, WFDC1, GHRL, TGFBR3, GHR, GNG7	9.5*E* − 05
GO:0006811	Ion transport	SLC36A1, SLC38A3, CASR, FXYD4, AQP1, FXYD6, BSND, SLC23A1, ATP6V0E2, P2RY4, ATP6V1E1, SLC39A8, SCN4B, SLC13A2, PLLP, SLC5A6, SLC4A1, ATP6V0D1	0.00018

*Downregulated miRNAs*			
GO:0009611	Response to wounding	CXCL1, MASP1, SCN3A, TACR1, CCR1, CXCL2, GJA1, TLR4, CCL7, TGFB2, TIMP1, VCAM1, PCSK1, CASP3, FGG, CD44, SERPINE1, IL1B, REG3G, KLF6, TNFSF4, LYN, MAP1B, IL1RN, C4BPA, IL24, P2RY12, FCGR2B, F3, CCR2, NPPB, IGFBP1, PTAFR, IGFBP4	2.0*E* − 16
GO:0006954	Inflammatory response	CXCL1, TNFSF4, LYN, MASP1, TACR1, CCR1, IL1RN, CXCL2, TLR4, C4BPA, CCL7, VCAM1, FGG, CD44, FCGR2B, F3, CCR2, IL1B, NPPB, REG3G, PTAFR, IGFBP4	1.6*E* − 12
GO:0006952	Defense response	CXCL1, MASP1, FGR, GRIK2, TACR1, CCR1, CXCL2, TLR4, CCL7, VCAM1, FGG, CD44, IL1B, REG3G, PTPRC, TNFSF4, LYN, IL1RN, C4BPA, FCGR2B, F3, CCR2, NPPB, PTAFR, IGFBP4	9.0*E* − 10

**Table 4:** KEGG pathway analysis of the predicted miRNAs targets.

KEGG pathway term	Target genes	*P* value
rno00280: valine, leucine, isoleucine degradation	BCKDHA, MCCC2, IVD, ECHS1, ACAT1, HIBADH, ALDH9A1	0.0018
rno04060: cytokine-cytokine receptor interaction	CCL22, CNTF, TNFSF4, IL9R, TNFRSF12A, CLCF1, CCR1, CXCL2, CCR2, IL21R, IL1B, FAS, GHR	0.010
rno04115: p53 signaling pathway	CASP3, CDKN1A, RRM2, CASP8, CYCS, SERPINE1, FAS	0.011
rno04540: gap junction	TUBB2B, TUBB5, GJA1, GUCY1A3, GUCY1B3, HTR2B, TUBB3	0.027
rno00380: tryptophan metabolism	CCBL1, CYP1A1, ECHS1, ACAT1, ALDH9A1	0.033
rno04080: neuroactive ligand-receptor interaction	ADORA2B, GRIK2, TACR1, GRIA3, GHRHR, P2RY4, SSTR1, CNR1, P2RY2, CCR2, HTR2B, PTAFR, ADRA1D, GHR	0.035
